# Development and Validation of a Spectrometric Method for Cd and Pb Determination in Zeolites and Safety Evaluation

**DOI:** 10.3390/molecules25112591

**Published:** 2020-06-02

**Authors:** Marin Senila, Oana Cadar, Ion Miu

**Affiliations:** 1 National Institute for Research and Development of Optoelectronics Bucharest INOE 2000, Research Institute for Analytical Instrumentation, 67 Donath Street, 400293 Cluj-Napoca, Romania; oana.cadar@icia.ro; 2 SC UTCHIM SRL, 12 Buda Street, 240127 Ramnicu Valcea, Romania; utchim_vl@yahoo.com

**Keywords:** zeolites, dietary supplements, cadmium, lead, GF-AAS, validation, risk assessment

## Abstract

An analytical method based on microwave-assisted acid digestion and atomic absorption spectrometry with graphite furnace as atomization source was developed and validated for determining trace elements (Cd and Pb) in zeolites used as dietary supplements, for their characterization and safety evaluation. The method was checked for the main performance parameters according to the legislation requirements in the field of dietary supplements. In all cases, the obtained performance parameters were satisfactory. The selectivity study showed no significant non-spectral matrix effect. The linearity study was conducted for the calibration curves in the range of 0–10 ng mL^−1^ for Cd and 0–30 ng mL^−1^ for Pb. The obtained limits of detection (LoDs) and the limits of quantification (LoQs) were sufficiently low in order to allow Pb and Cd determination in dietary supplements. For the internal quality control, certified reference materials were analysed and good recoveries were obtained. The precision study was performed in terms of repeatability and reproducibility, considering the requirements imposed by the Commission Decision (2007/333/EC) and the method fulfilled these performance parameters. Expanded measurement uncertainties were estimated to 11% for Cd and 10% for Pb. Cd and Pb content were measured in real zeolite samples and, using these data, a safety evaluation was carried out.

## 1. Introduction

Natural zeolite tuffs are crystalline materials with excellent properties, for example high adsorption capacity, ion-exchange ability, catalyzing action, and thermal stability. The chemical structure of zeolites contains silicon and aluminum interlinked through one, two, or three oxygen atoms, which leads to a wide diversity of three-dimensional potential structures. Zeolites have a negative charge balanced by positively charged monovalent and divalent ions of alkali and alkali earth elements such as water molecules that can be easily substituted by other cations or molecules. The Si/Al ratio in a zeolite has influence on its negative charge and on the attraction of the foreign ions inside their pores and channels [1,2]. The natural zeolites are mostly used in water treatment, catalysis, petrochemistry, cosmetics, agronomy, and medicine [3,4].

About 70 types of natural zeolites have been identified around the world with the most common mineral that occurs in natural zeolites being clinoptilolite. The clinoptilolite possess high surface area, good adsorption capacity, chemical and thermal stability, and ion-exchange capacity, which make it a potential detoxifying agent for organisms and a support in many medical applications. Clinoptilolite was found to be efficient in the veterinary and human medicine [5]. In the last years, the clinoptilolite-based zeolite is increasingly studied for use in human medicine as dietary supplements. Basha and co-workers combined clinoptilolite with other substances such as EDTA in order to increase the detoxifying capacity [6]. Federico and co-workers used micronized clinoptilolite for adsorption of ingested ethanol in order to reduce the blood alcohol level [7].

Zeolites attract trace metals in their structure, which is a property that makes them useful to eliminate these elements from the human body [8]. However, in the natural environment, this property may cause the accumulation of toxic metals in natural zeolites. The occurrence and the concentration of toxic metals in natural zeolite minerals vary depending on the quarry [9].

Cd and Pb have no known biological functions, and are classified as non-essential elements that are toxic even at very low concentrations for human health [10,11,12]. Cd is associated with negative health effects on renal, skeletal, pulmonary, reproductive, and cardiovascular systems, which are classified as group I carcinogens to humans [13]. Pb may also causes serious damages for human health and may cause even death [14,15,16]. The presence of toxic metals in the raw ingredients used to produce dietary supplements is undesirable. Therefore, the quality of both raw materials and end products must be strictly controlled [17].

Considering the high toxicity of both metals, the maximum Cd and Pb concentrations in dietary supplements of 1.0 mg kg^−1^ and 3.0 mg kg^−1^, respectively were established by legislation in the field [18,19]. These regulations impose to validate sensitive methods that provide trusting results even at low level of concentration, having well-established performance criteria [20,21].

Three groups of analytical techniques are the most used in the routine laboratories to measure metals in different liquid and solid samples. The main differences among these techniques consist in the detection of analyte and type of atomization source. The first group uses a mass spectrometer to measure ions of analyte produced using inductively coupled plasma (ICP-MS) [22]. In the second group of analytical techniques, the optical emission of analyte ions is measured at specific wavelengths in an inductively coupled plasma (ICP-OES) [23,24]. The third group of atomic spectrometric techniques is based on the absorption of radiation at a specific wavelength by the atoms of analyte. In this case, the atoms can be obtained by electrothermal evaporation in a graphite furnace (GF-AAS) or in a flame (F-AAS) [25,26,27]. Beside these, other non-conventional techniques can also be used to determine metal analytes in different samples [28,29,30]. For specific elements (*i.e.,* Hg) which have high volatility, the direct measurement in solid samples (including zeolites) by thermal desorption is possible [31].

Although the market growth of dietary supplements based on zeolites, no validated analytical methods for the analysis of Pb and Cd in zeolites was found in literature. Currently, the GF-AAS technique is widely used for its recognized high selectivity and sensitivity. Due to these technical features, our study focuses to perform a fully-validation of this method in view of the legislative demands for dietary supplements [32], in order to produce reliable analytical data. In addition, the concentrations of both trace metals were measured in real zeolite samples to assess if the intake of zeolites as dietary supplements represents a risk for human health.

## 2. Results and Discussion

### 2.1. Performance Parameters

The main performance parameters such as selectivity, working and linear ranges, LoD and LoQ, precision, accuracy, and measurement uncertainty were evaluated for validation of the method for determination of Cd and Pb in zeolite samples [33,34,35]. The targets imposed for these parameters considered the quality standards for analytical methods provided by Commission of the European Communities in Regulation No. 333/2007 [20].

A graphite furnace atomic absorption spectrometer with graphite atomizer Perkin Elmer model PinAAcle 900T (Norwalk, CT, USA) was used to determine Cd and Pb in digested samples using direct injection of samples, followed by addition of an appropriate matrix modifier, as recommended by the instrument manufacturer. The calibration standards were prepared by the instrument autosampler by diluting the highest concentrated standard solution (10 ng mL^−1^ for Cd and 30 ng mL^−1^ for Pb) with ultrapure water. Electrodeless Discharge Lamps (EDLs) were used as spectral sources. The instrument operating conditions used are presented in Table 1.

#### 2.1.1. Selectivity

**Figure 1:**
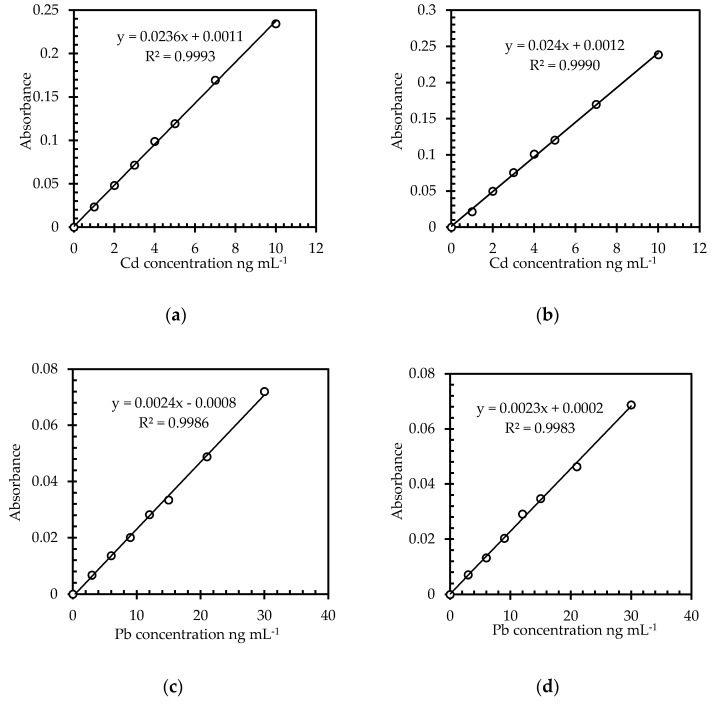
Calibration curves for Cd and Pb determination by graphite furnace atomic absorption spectrometry (GF-AAS): (**a**) Cd prepared in 0.5% (*m*/*v*) HNO_3_, (**b**) Pb prepared in 0.5% (*m*/*v*) HNO_3_, (**c**) Cd prepared in complex matrix that mimic the solution of digested samples, and (**d**) Pb prepared in complex matrix that mimic the solution of digested samples.

To provide trustable results, an analytical method should differentiate the analytes of interest, in this case Cd and Pb, from other compounds in the sample matrix [33]. In order to improve the selectivity, for some elements including Cd and Pb, matrix modifiers are used in the GF-AAS technique to decreases the volatility of the analytes and to prevent their loss during thermal decomposition. The estimation of the matrix effect on the analytical signal is of high importance to assure that the most suitable calibration method was selected. The digested zeolite sample contains important amounts of elements dissolved from the solid sample and high content of mineral acids (HNO_3_, HCl, HF, and H_3_BO_3_) used for digestion, thus the analyte signal from the sample might differ from the signal of the same analyte concentration in aqueous calibration standards. The selectivity was evaluated based on the matrix effects produced by the high amounts of Al, Ca, Mg, K, Na, and Fe in the digested zeolite solution. In order to evaluate the matrix effects, two calibration standards were constructed for both Pb and Cd. A calibration standard of 10 µg L^−1^ Cd and a calibration standard of 30 µg L^−1^ Pb were prepared in 0.5% (*m/v*) HNO_3_, while other standards of 5 µg L^−1^ Cd and of 20 µg L^−1^ Pb were prepared in a solution containing 2.0% (*m/v*) HNO_3_, 3.0% (*m/v*) HCl, 0.8% (*m/v*) HF, and 1.0% (*m/v*) H_3_BO_3_ as well as concentrations of 250 mg L^−1^ Al, 100 mg L^−1^ Ca, 40 mg L^−1^ Mg, 40 mg L^−1^ K, 20 mg L^−1^ Fe, and 20 mg L^−1^ Na to mimic the solution of digested samples. Ratios of the slopes for both types of calibration curves were calculated, and the value was compared with 1, to find out if positive or negative influence on the analytes signals is given by interfering components. Figure 1 presents the plots of analytical signals for Cd and Pb against their concentrations using the two sets of calibration standards.

Comparison of the curves’ slopes by means of their ratio revealed no significant influence of the matrix effect since the ratio were 1.02, and 0.96 for Cd and Pb, respectively. Consequently, calibration solutions prepared in 0.5% (*m*/*v*) HNO_3_ were used for calibration in all experiments.

#### 2.1.2. Linearity Study

The linear regression analysis was applied for all the calibration curves built using diluted nitric acid and complex matrix that mimic the digested zeolite solution. The calibration curves were prepared by including the allowed Residual Maximum Limit (RML) in the middle of their ranges by taking into account a factor of 200 (due to the digestion of 0.5 g sample and dilution to a volume of 100 mL), and the instrument sensitivity for each element. Thus, for Cd, the concentrations of the calibration curve were chosen in the range of 0–10 ng mL^−1^ since the RML for Cd in food supplements, according to Decision 2008/629/EC [18], is 1.0 mg kg^−1^. Considering the factor of 200, due to the sample digestion, this concentration of 1.0 mg kg^−1^ corresponds to a concentration in solution of 5.0 ng mL^−1^. Similarly, for Pb, a concentration in solution of 15 ng mL^−1^ was calculated since it has a RML in food supplements of 3.0 mg kg^−1^, and, consequently, the range of concentration was 0–30 ng mL^−1^. Seven levels of concentration were used for the calibration of each element (0.2, 0.4, 0.6, 0.8, 1.0, 1.4, and respectively 2 times RML). The correlation coefficients (r) for Cd and Pb, respectively, for both calibration aqueous standards and standards with matrix, as presented in Figure 1, fulfill the requirements of r > 0.9950.

The test for homogeneity of variances of the linear calibration curves was done according to the requirements of ISO 8466-1 [36]. Ten replicates of the lowest (0.2 RM) and 10 of the highest (2 RML) concentrations prepared for the calibration curves were measured. The square of the standard deviations (s) for the two concentrations were calculated and the testing values s^2^_2MRL_ / s^2^_0.2MRL_ = 3.31 for Cd and s^2^_2MRL_ / s^2^_0.2MRL_ = 4.40 for Pb were compared with the Fisher–Snedecor distribution value for *n* = 9 degrees of freedom and 0.99 probability (F = 5.35). In both cases, the testing values were below than the F distribution value indicating a satisfactory homogeneity of variances for the chosen ranges of calibration curves.

The working ranges for the solid samples are limited at the lower part while, for the upper part, these concentrations are 2.0 mg kg^−1^ for Cd and 6.0 mg kg^−1^ for Pb, which are calculated from the sample preparation step and from the calibration curve ranges. Higher concentrations can be measured by dilution of samples.

#### 2.1.3. Evaluation of LoDs and LoQs

Being the lowest content of an analyte that can be detected in a sample, LoD was calculated as the ratio between three times standard deviation of the mean resulted by measuring the blank signal (*n* = 21) and the slope obtained for calibration curves [20].

The LoD for Cd in digested liquid sample was calculated to have the value of 0.15 ng mL^−1^, which corresponds to a concentration in a solid sample of 0.03 mg kg^−^^1^. For Pb, LoD in a digested liquid sample was found to be 0.64 ng mL^−1^, which means 0.13 mg kg^−1^ in a solid sample. These values were in agreement with the demands of Commission Regulation (EU) No 836/2011 [37] where, for RML ≥ 0.100 mg kg^−1^, LoDs should be ≤ one tenth of the RML (1.0 mg kg^−1^ for Cd and 3.0 mg kg^−1^ for Pb). Our values were similar with those reported by Ivanova-Petropulos et al. [25] when analyzed water and brandy by GF-AAS (LoD of 0.12 ng mL^−1^ and 0.27 ng mL^−1^ for Cd, and of 0.57 ng mL^−1^ and 0.68 ng mL^−1^ for Pb). Aleluia et al. [38] reported LoDs in the same order of magnitude for Cd and Pb determination in organic pharmaceutical formulations using high-resolution continuum source GF-AAS.

LoQ is the lowest concentration of analyte that can be measured with acceptable precision and accuracy. The ten times standard deviation from the measuring the blank signal (*n* = 21) divided to the slope of calibration curves was used to calculate the LoQs [20,39]. The calculated LoQ for Cd was 0.50 ng mL^−1^, corresponding to a concentration in a solid sample of 0.10 mg kg^−1^, while, for Pb, the calculated LoD was 2.13 ng mL^−1^, corresponding to a value of 0.43 mg kg^−1^ in the solid sample. These values are below the imposed limits of legislative requirements [37] for RML ≥ 0.100 mg kg^−1^, where LoQs should be ≤ one fifth of the RML for Cd and Pb. The found values were in similar range with published data in previous studies [11,38].

Confirmation of LoQs was carried out by analysing series of ten spiked solutions with Cd and Pb content levels of 0.50 ng mL^−1^, and, respectively, 2.00 ng mL^−1^. The relative standard deviations of repeatability (RSDr%) were 10.4% (Cd), 12.9% (Pb), while the recoveries for that levels of concentration were 88% (Cd)and 94% (Pb). The obtained results were within the range of the imposed targets (RSDr% <20% and a recovery rate of 85–115%).

#### 2.1.4. Trueness and Precision

The trueness was evaluated by analysing certified reference materials (CRMs) with a similar matrix to zeolite samples. Six replicates of both CRMS: potassium feldspar NIST-SRM 70b and Loam soil ERM – CC141, were analysed by GF-AAS under similar conditions as the real samples. The certified values of CRMs, measured values, and the recoveries degree (%) are offered in Table 2.

The results obtained for recoveries degree in CRMs for both Cd and Pb were situated in the acceptable range of 80–110%. This range was chosen considering the demands of the Commission Decision 2002/657/EC [32].

The precision study was performed in terms of repeatability and internal reproducibility while taking into account the requirements imposed by the legislation in the field of dietary supplements [20,37] for these performance parameters. The RSD of repeatability (RSD_r_) was assessed by analyzing in parallel six aliquots of fortified samples at concentrations levels of 0.2 and respectively 1 times RML, using the same equipment on the same day. The same concentration levels of fortified samples were analysed on different days to calculate RSD of of reproducibility (RSD_R_). For the acceptance criteria, the HorRat indexes were considered. HorRat index can be obtained by dividing the RSD_r_ or RSD_R_ to a predicted value of RSD (PRSD) calculated using Horvitz’s equation [40]. PRSD depends on the mass fraction of the analyte in the sample

Using the Horvitz’s equation, we calculated the PRSD for a mass fraction of 1000 µg kg^−1^ (RML of Cd) equal to 16%, while, and, for a mass fraction of 3000 µg kg^−1^ (RML of Pb), a PRSD equal to 14%. HorRat_r_, HorRat_R_ indexes, together with the PRSD calculated for 0.2 RML and 1 RML levels of concentrations for both Cd and Pb are presented in Table 3.

In all cases, the HorRat_r_, HorRat_R_ indexes were below the maximum value of 2, as required in legislation in the field [37]. Thus, according to the obtained results in the repeatability and reproducibility study, the method for Cd and Pb analysis of zeolite samples using GF-AAS is sufficient precise.

#### 2.1.5. Measurement Uncertainty

Regarding the value of measurement uncertainty for analytical methods designed for dietary supplement analysis, there are imposed limits that depend on the calculated LoD for that specific analyte [37]. The maximum standard uncertainty is calculated considering LoD, the concentration of the analyte and a numeric factor (α) that is determined by the RML of that analyte in sample (α = 0.15 for Cd RML at a mass fraction of 501–1000 µg kg^−1^, and α = 0.12 for Pb RML at a mass fraction of 1001–10000 µg kg^−1^).

For a RML for Cd of 1000 µg kg^−1^, the maximum uncertainty of measurement for Cd was calculated to be 150 µg kg^−1^ (maximum relative standard uncertainty, U_rel_ = 15%). For Pb, that have RML of 3000 µg kg^−1^, the maximum uncertainty of measurement (U_f_) was found equal to 360 µg kg^−1^, (maximum relative standard uncertainty, U_rel_ = 12%).

The measurement uncertainty is evaluated by combining different identified main sources [41]. These can be identified and quantified in the validation process and can be obtained from calibration certificates of volumetric flacks, pipettes, measuring instruments (declared uncertainty) and from the repeated measurements in the trueness and repeatability studies during the method validation.

It was assumed that the validation study comprises the total analytical procedure, and, thus, the most relevant uncertainty components that influence the expanded measurement uncertainty were assembled into only repeatability and accuracy components, that can be obtained from analysis of CRMs. Standard uncertainty is evaluated by combining the standard deviation of multiple determinations on CRM and the difference between found values and certified values of CRM.

The expanded uncertainty (U) is obtained from the standard uncertainty and a cover factor that depends on the level of confidence, *P* (*k* = 2, for *p* = 95%). In case of Pb, two CRMs were analysed. Thus, for the whole method a pooled expanded uncertainty (%) was calculated by combining the two expanded uncertainties.

The expanded uncertainty (%) for Cd was calculated to be 11%, while the expanded uncertainty (%) for Pb was found to be 10%. These results are within the maximum value of 15% for Cd and 12% for Pb established according to the legislative demands, and are considered satisfactory values.

### 2.2. Analysis of Real Samples

Five samples were used in the study including two commercial dietary supplements of micronized natural zeolite samples (S1 and S2) and three zeolite tuff samples (P1, P2, and P3) collected from different quarries from North-west Romania.

#### 2.2.1. Zeolite Chemical and Mineralogical Characterization

**Figure 2:**
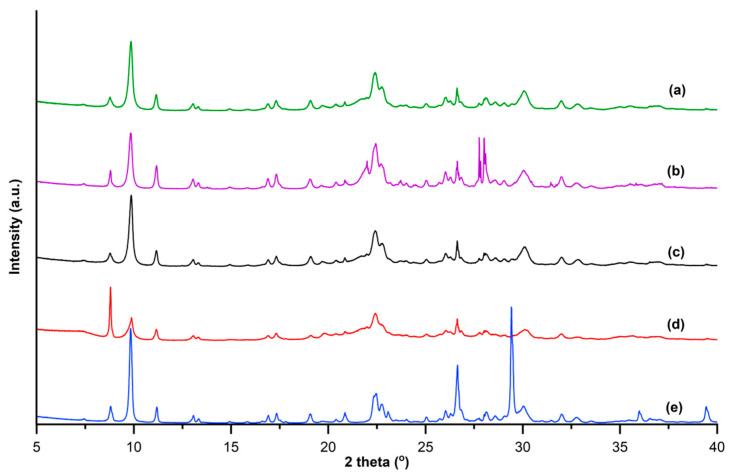
The pXRD patterns of (**a**) S1, (**b**) S2, (**c**) P1, (**d**) P2, and (**e**) P3 zeolites.

All pXRD patterns of investigated zeolite samples (show the characteristic peaks of clinoptilolite at 2θ values of 9.86°, 11.16°, 22.46°, 26.03°, and 31.95° (Figure 2) [42]. According to pXRD analysis, the zeolite samples contain clinoptilolite as a major crystalline phase, which is accompanied by quartz, muscovite, feldspar, montmorillonite, and albite. The P3 sample also contains traces of calcite.

The samples were characterized regarding the chemical composition by measuring Al, Fe, Na, K, Ca, Mg, and Ti concentrations using ICP-OES. Then, using the atomic masses were the mass fraction the values were transformed in oxides content. SiO_2_ content was obtained using the gravimetric method. The measured oxide concentrations are presented in Table 4.

The chemical composition of samples confirms the pXRD analysis since clinoptilolite-type zeolite has a Si to Al ratio higher than 4 and a content of Na + K higher than Ca content [43]. Thus, according to these data, in all samples, the main mineral is clinoptilolite.

#### 2.2.2. Cd and Pb Determination in Zeolite Samples by GF-AAS Technique after Microwave-Assisted Acid Digestion

The results obtained for Cd and Pb determination for real samples of dietary supplements and zeolites quarried from NW Romania measured using the GF-AAS technique and, for comparison, using the inductively coupled plasma mass spectrometry (ICP-MS) technique, are presented in Table 5. The Cd concentrations in all investigated samples were below the LoQ (0.1 mg kg^−1^). Thus, from this point of view, both dietary supplements and possible use of zeolite tuffs from investigated quarries as raw material for food supplements are safe for human health. In case of Pb, in samples of dietary supplements, it was found but its concentration was lower than the maximum limit of 3.0 mg kg^−1^ imposed by Decision 2008/629/EC. In case of all samples collected from the quarries, the maximum admitted value established by legislation was exceeded. A review analysis carried out by de Vasconcelos Neto et al. [16] on the Pb contamination in food, generally showed lower concentrations than in the present study. However, for zeolites samples from Serbia, Tomasevic-Canovic [9] reported Pb concentrations in the range of 29–38 mg kg^−1^, which is higher than in our case. Thus, there is a need to measure toxic metals in this type of samples. As presented in Table 5, the accuracy of GF-AAS technique was also proved by similarity of the results obtained using this with those obtained by ICP-MS technique.

#### 2.2.3. Risk Exposure

The risk exposure to Pb through the consumption of zeolite dietary supplements was evaluated taking into account the provisional tolerable weekly intake (PTWI) [44,45]. The weekly intake (mg kg^−1^ b.w.) was evaluated for an usual intake of four pills containing zeolite supplements per day in which each of them are 0.35 g (a consumption of 9.8 g of zeolite supplemented per week) for a 60 kg adult. The equivalent weekly risk exposure to Pb was assessed as a percentage of PTWI considering the Pb PTWI value of 0.025 mg kg^−1^ b.w., as recommended by WHO [46].

Considering this consumption, for an average concentration of 2.4 mg kg^−1^ as found in dietary supplements, % PTWI = 1.6%, which is much lower than the imposed Pb PTWI. Even in the worst case of use of raw material zeolite containing 15 mg kg^−1^ Pb, the percentage of PTWI = 9.8% (≅10%) from the recommended value for Pb of WHO. This is because the mass of the consumed supplement is relatively small and does not significantly influence the intake of Pb. However, the maximum limit of 3.0 mg kg^−1^ imposed by Decision 2008/629/EC should be considered when using zeolite as a dietary supplement. Cd was not considered in the assessment of risk exposure, since, in all cases, its concentrations in analysed samples were below LoQ.

This study is of considerable interest since: (*i*) to our knowledge, no validated analytical methods for Cd and Pb measurement in zeolites samples are presented in literature, (*ii*) provides the necessary steps to validate a method in agreement with the demands of legislation, and (*iii*) no previous data were reported on the literature on the risk exposure to Pb via consumption of zeolite dietary supplements.

## 3. Materials and Methods

### 3.1. Standard Solutions, Reagents, and CRMs

All the solutions were prepared using ultrapure water produced by a Milli-Q system Direct Q3 (Millipore, Molsheim, France). Analytical-grade reagents (65% HNO_3_, 40% HF, 37% HCl, and 99.5–100.5% H_3_BO_3_) used in experiments were purchased from Merck (Darmstadt, Germany). Mono-element Cd and Pb calibration standards for GF-AAS were prepared by sequential diluting stocks solutions (Merck, Darmstadt, Germany) containing 1000 mg L^−1^ of these elements in 0.5% (*v*/*v*) HNO_3_. For the ICP-OES calibration, standard solutions in the range of 0–20 mg L^−1^ were prepared from 1000 mg L^−1^ multi-elements containing elements of interest of 1000 mg L^−1^ and mono-element (Ti) standard solution 1000 mg L^−1^ (Merck, Darmstadt, Germany), diluted in 0.5% (*m*/*v*) HNO_3_.

Matrix modifiers of 10% NH_4_H_2_PO_4_, 1% MgNO_3_ (Perkin Elmer Pure, Shelton, DC, USA) were used to prepare chemical modifier solution containing the mixture of 0.1% (*m*/*v*) Pd plus 0.05% (*m*/*v*) Mg in 0.5% (*v*/*v*) HNO_3_. Argon (5.0 quality) from Linde Gas SRL Cluj-Napoca, Romania was used for instruments.

Potassium feldspar Standard Reference Material (NIST-SRM 70b) produced by the National Institute of Standards & Technology USA and Loam soil ERM–CC141 produced by the Institute for Reference Materials and Measurements and purchased from LGC Promochem (Wesel, Germany) were used.

### 3.2. Instrumentation and Methods

A GF-AAS model PinAAcle 900T (Perkin Elmer, Norwalk, CT, USA) was used for Cd and Pb determination. An inductively coupled plasma mass spectrometer ICP-MS ELAN DRC II (Perkin-Elmer, Toronto, ON, Canada) was used to measure Cd and Pb for a comparison of results with those obtained by GF-AAS. The content of major elements was determined by ICP-OES using an SPECTROFLAME FMD-07 (Spectro Analytical Instruments, Kleve, Germany). The conversion to the corresponding oxide was made by multiplying the element concentration with 1.8895 (Al_2_O_3_), 1.4297 (Fe_2_O_3_), 1.3392 (CaO), 1.6583 (MgO), 1.2046 (K_2_O), 1.3480 (Na_2_O), 1.2912 (MnO), and 1.6683 (TiO_2_). SiO_2_ content was measured by a gravimetric method.

Microwave digestion of a 0.5 g powder of sample using microwave-assisted acid digestion using a microwave system (Berghof, Eningen, Germany) was described elsewhere [31]. The heating program of the microwave system for samples digestion comprised three steps of heating at 160 °C, 200 °C, the cooling at 100 °C, in a total time of digestion of 35 min. After cooling at room temperature, 20 mL of saturated H_3_BO_3_ were added and the samples were heated again at 160 °C in the microwave system for 15 min.

The samples were cooled down to room temperature and filtered on cellulose filters (circles, diameter of 125 mm) (Whatman, Germany) using PTFE funnels in volumetric flasks of 50 mL and diluted to final volume using ultrapure water. The resulted solution was analyzed by GF-AAS for Cd and Pb and by ICP-OES for Na, K, Ca, Mg, Fe, Al, and Ti, respectively.

The powder X-ray diffraction (pXRD) patterns were recorded at room temperature, using a D8 Advance (Bruker, Karlsruhe, Germany) diffractometer, operating at 40 kV and 40 mA with CuK_α_ radiation (λ = 1.54060 Å), at room temperature.

### 3.3. Real Samples Preparation

Two commercial dietary supplements of micronized natural zeolite samples (S1 and S2) and three zeolite tuff samples collected from different quarries from North-West Romania (P1–Racos, Brasov County, P2–Chilioara, Salaj County, and P3–Macicas, Cluj County) were used in this study. The samples were collected from the quarries as rock, then crushed, then grounded to a fine powder. The powder of each individual sample was further micronized using a laboratory micronization unit Pilotmill-2 (FPS, Como, Italy).

### 3.4. Strategy for Method Validation

The GF-AAS method was validated for the analysis of Cd and Pb in zeolites used as dietary supplements in terms of matrix effect, selectivity, linearity, working range, LoD and LoQ, trueness, precision, and measurement uncertainty. The performance parameters were assessed in comparison with the target limits established by legislation in the field of food control and dietary supplements analysis [20,32].

### 3.5. Risk Exposure

The risk exposure to Pb through the ingestion of zeolite dietary supplements was evaluated using a methodology to assess the exposure to a contaminant based on a provisional tolerable weekly intake. The equivalent weekly risk exposure to Pb was calculated as the percentage of PTWI by considering the Pb PTWI value of 0.025 mg kg^−1^ b.w., as suggested by WHO [46].

%PTWI =100 × Estimated exposure to Pb/Pb PTWI(1)

where Pb PTWI = 0.025 mg kg^−1^ b.w.

## 4. Conclusions

In this paper, an analytical method based on the microwave-assisted acid digestion and GF-AAS technique for determining Cd and Pb in zeolites is validated, considering the demands of European legislation for the official control of trace elements in food supplements. The obtained LoDs (0.03 mg kg^−^^1^ for Cd and 0.13 mg kg^−^^1^ for Pb) and LoQ (0.10 mg kg^−^^1^ for Cd and 0.43 mg kg^−^^1^ for Pb) were lower than the imposed limits of specific legislation. The trueness was evaluated by analysing two certified reference materials, NIST-SRM 70b and ERM–CC141. Good recoveries were obtained. The precision study was performed in terms of repeatability and internal reproducibility, considering the requirements imposed by the Commission Decision (2007/333/EC). The expanded measurement uncertainties were estimated to 11% for Cd and 10% for Pb and fulfill the legislative requirements. The content of Cd and Pb was measured in several zeolite samples. Using these data, a safety evaluation was carried out. It was concluded that, considering the level of Cd and Pb concentrations and the estimated weekly intake of zeolite as dietary supplements, their consumption does not pose a risk for human health.

## Figures and Tables

**Table 1:** Operation conditions for Cd and Pb determination in zeolites by graphite furnace atomic absorption spectrometry (GF-AAS).

**All elements**				
Signal processing	Peak area			
Read time	5 s			
Sample volume	20 µL			
Background correction	Longitudinal Zeeman-effect			
**Cd**				
Wavelength	228.80 nm			
EDL current	230 mA			
Calibration	0–10 ng mL^−1^ (7 points)			
Matrix modifier	50 µg NH_4_H_2_PO_4_ + 3 µg Mg(NO_3_)_2_ (5 µL)			
**Furnace Program**				
**Step**	**Temp (°C)**	**Ramp (s)**	**Hold (s)**	**Ar Flow-Rate (mL min^−1^)**
Drying	110	1	40	250
Drying	130	15	40	250
Ashing	500	10	20	250
Vaporization	1500	0	5	0
Cleaning	2450	1	3	250
**Pb**				
Wavelength	283.31 nm			
EDL current	400 mA			
Calibration	0–30 ng mL^−1^ (7 points)			
Matrix modifier	50 µg NH_4_H_2_PO_4_ + 50 µg Mg(NO_3_)_2_ (5 µL)			
**Furnace Program**				
**Step**	**Temp (°C)**	**Ramp (s)**	**Hold (s)**	**Ar Flow-Rate (mL min^−1^)**
Drying	110	1	40	250
Drying	130	15	40	250
Ashing	850	10	20	250
Vaporization	1600	0	5	0
Cleaning	2450	1	3	250

**Table 2:** Certified values of CRMs, measured values (*n* = 6 parallel determinations) and the recoveries degree (%).

CRM	Certified Values ± U ^a^ (mg kg^−1^)		Measured Values ± U ^b^ (mg kg^−1^)		Recovery (%)	
	Cd	Pb	Cd	Pb	Cd	Pb
NIST-SRM 70b	-	57 ± 3.0	-	55 ± 5.2	-	96
ERM–CC141	0.35 ± 0.05	41 ± 4.0	0.32 ± 0.035	43 ± 4.6	91	105

^a^: U = expanded uncertainty (*k* = 2); ^b^: U = calculated expanded uncertainty (*k* = 2)

**Table 3:** Calculated PRSD, HorRat_r_, HorRat_R_ indexes.

Mass Fraction	Repeatability Study		Reproducibility Study		Results
	PRSD	HorRat_r_	PRSD	HorRat_R_	
0.2 RML Cd (200 µg kg^−1^)	20	0.55	20	0.60	Admitted
1 RML Cd (1000 µg kg^−1^)	16	0.50	16	0.44	Admitted
0.2 RML Pb (600 µg kg^−1^)	17	0.65	17	0.82	Admitted
1 RML Pb (3000 µg kg^−1^)	14	0.60	14	0.64	Admitted

**Table 4:** Mass fraction in oxides (%) in real samples.

Compounds	Concentrations (%)				
	S1	S2	P1	P2	P3
SiO_2_	67.5	69.5	58.9	61.4	68.3
Al_2_O_3_	10.8	11.4	12.0	12.2	9.42
CaO	2.32	2.88	2.67	2.93	1.91
MgO	0.89	0.46	1.55	1.47	0.76
K_2_O	2.34	2.47	2.20	2.03	2.96
Na_2_O	1.07	0.41	0.87	0.44	0.29
TiO_2_	0.20	0.18	0.13	0.12	0.19
Fe_2_O_3_	1.05	0.47	1.58	0.89	0.81

**Table 5:** Concentrations of total Cd and Pb (mg kg^−1^) measured in zeolite samples by GF-AAS and ICP-MS after sample digestion (*n* = 6 parallel measurements). Legend: – results presented as mean ± expanded uncertainty, <0.10 and <0.05–below limits of quantification (LoQ) for GF-AAS and ICP-MS, respectively.

Zeolite Sample	Cd (mg kg^−1^)		Pb (mg kg^−1^)	
	GF-AAS	ICP-MS	GF-AAS	ICP-MS
S1	<0.10	<0.05	2.6 ± 0.3	2.4 ± 0.3
S2	<0.10	<0.05	2.1 ± 0.3	2.0 ± 0.2
P1	<0.10	<0.05	8.2 ± 0.5	8.5 ± 0.8
P2	<0.10	<0.05	22 ± 2	23 ± 3
P3	<0.10	<0.05	15 ± 2	14 ± 2
